# Detection and characterization of *Clostridium botulinum* isolated from powdered infant formula

**DOI:** 10.3389/fmicb.2026.1800624

**Published:** 2026-06-15

**Authors:** Cesar Nadala, Eni Themeli, Fereidoun Forghani, Youngsil Ha, Sukkyun Han, Anna Shapovalova, John Roach, Seong Hong Kim, Jennifer L. M. Thorson, Joo-Young Lee, Matthew Spear, Benjamin Buhrman, Kevin Chiu, Niall Mullane, Mansour Samadpour

**Affiliations:** 1 IEH Laboratories and Consulting Group, Lake Forest Park, WA, United States; 2 ByHeart, New York, NY, United States

**Keywords:** *Clostridium botulinum*, food safety, infant botulism, outbreak investigation, powdered infant formula (PIF), sulfite-reducing clostridia (SRC), whole-genome sequencing (WGS)

## Abstract

**Introduction:**

As a part of an ongoing investigation of the 2025 multistate outbreak of infant botulism linked to consumption of powdered infant formula (PIF), various samples – including unopened containers of PIF and base powder (bulk PIF before packaging) – were analyzed for the presence of *Clostridium botulinum*.

**Methods:**

Samples were screened using a tiered analytical approach: anaerobic enrichment followed by real-time PCR targeting botulinum neurotoxin-associated genes, confirmatory PCR, amplicon sequencing, colony isolation, and whole-genome sequencing (WGS). *C. botulinum* was detected in both the finished product and base powder.

**Results:**

Genomic analysis revealed genetic identity between isolates from one finished product lot and a base powder, while an isolate from another lot was distinct. Importantly, detection occurred in samples with non-detectable sulfite-reducing clostridia (SRC).

**Conclusion:**

Results of this study demonstrate that indicator-based screening (such as SRC enumeration) even if it had been in place before the outbreak, would not have prevented its occurrence. Positive linkage of an infant botulism outbreak to PIF should compel manufacturers to recognize *C. botulinum* as a hazard reasonably likely to occur in certain ingredients, necessitating the design and implementation of specific preventative controls.

## 1 Introduction

*Clostridium botulinum* is an obligate anaerobic, spore-forming bacterium capable of producing botulinum neurotoxins (BoNT), which are among the most potent biological toxins known. These neurotoxins cause botulism, a rare but severe paralytic illness that presents a particular risk to infants, whose immature gut microbiota and physiological conditions allow intestinal colonization and *in situ* toxin production (Centers for Disease Control and Prevention (CDC), 1998; Lindström and Korkeala, 2006; Harris and Dabritz, 2024). Infant botulism is most associated with environmental exposure to spores originating from soil and dust; however, additional exposure pathways remain of concern given the extreme toxicity of BoNT and the low infectious dose required for disease (Hauschild and Dodds, 1993; Peck, 2009).

PIF has historically been considered a potential vehicle of concern for spore-forming bacteria because of its low water activity, extended shelf life, and the inherent resistance of clostridial spores to desiccation and other environmental stresses (American Public Health Association, 2015; Hauschild and Dodds, 1993; Doyle and Buchanan, 2013). Despite the theoretical considerations, the prevailing view and the advice given to the industry in recent years has been that *C. botulinum* is not a hazard reasonably likely to occur in PIF manufacturing.

In a 2004 Joint FAO/WHO Expert Meeting on Microbiological Risk Assessment, *C. botulinum* was classified as a microorganism capable of causing illness in infants but not identified as a hazard in PIF (Joint FAO/WHO Expert Meetings on Microbiological Risk Assessment (JEMRA) and World Health Organization, 2004). Consequently, *C. botulinum* is not regarded as a hazard in the Codex Code of Hygienic Practice for Powdered Formulae for Infants and Young Children (Codex Alimentarius Commission and Food and Agriculture Organization of the United Nations, 2008). Building on this assessment, the International Commission on Microbiological Specifications for Foods (ICMSF) (2014) recommended the use of SRC enumeration as a general indicator of anaerobic spore contamination, rather than routine testing for clostridial spores or *C. botulinum*, reflecting the presumed low prevalence of the organism in infant formula.

Nevertheless, the severe clinical consequences of botulism and the heightened vulnerability of the infant population necessitate continued vigilance. Within this context, the detection, isolation, and characterization of *C. botulinum* from infant formula–associated matrices provide valuable insights into contamination pathways, organism persistence, and the limitations of current detection strategies (Peck, 2009; Popoff and Bouvet, 2013).

In 2025, a multistate outbreak of infant botulism in the United States prompted recalls of PIF products^1^. As part of the associated investigation, IEH Laboratories and Consulting Group was engaged to screen, detect, and characterize *C. botulinum* from finished products, using a tiered analytical approach combining anaerobic enrichment, molecular screening, culture confirmation, and genomic analysis.

## 2 Materials and methods

### 2.1 Enumeration assays

A 25 g portion of sample was enumerated for aerobic and anaerobic mesophilic spores according to the Compendium of Methods for the Microbial Examination of Foods (Fifth edition). Sulfite reducing clostridia were enumerated according to ISO 15213-1:2023 (2023).

### 2.2 Enrichment and qPCR

A 100 g portion of the sample was enriched for botulinum-producing clostridia (spores and vegetative cells) by germination and growth in tryptone-peptose-glucose-yeast extract (TPGY) broth under anaerobic conditions following ISO/TS 17919:2013 (2013) protocol with minor modifications such as larger sample size. All samples were subjected to heat shock at 70–80 °C for 10–12 min before enrichment. After enrichment, a 1 mL aliquot of enrichment media was subjected to DNA purification using silica coated magnetic beads and the DNA eluted in 50 μL of sterile deionized water. A 2 μL aliquot of eluted DNA was tested using a *C. botulinum* BoNT/A and BoNT/B Real-Time PCR assay. The assay uses primers and probes for BoNT/A and BoNT/B as specified in ISO/TS 17919:2013 (2013) Annex C. The assay targets BoNT/B (FAM), BoNT/A (HEX), and *C.* spp. 16S (ROX) ribosomal RNA genes, as well as an internal control target in the Cy5 channel. PCR cycling was as follows: 95 °C, 5 min; 40 cycles of 95 °C, 20 s, 55 °C, 20 s, 72 °C, 20 s.

### 2.3 Confirmation of the presence of *C. botulinum*

Presumptive positive enrichment cultures were subjected to multiple PCR amplifications using the FDA botA983 primer set (U.S. Food and Drug Administration (FDA), 2025), an in-house botA782 primer set, and the FDA botB492 primer set (U.S. Food and Drug Administration (FDA), 2025). PCR amplicons were purified using silica-based spin columns and sequenced on a Flongle flow cell using the MinION Mk1C platform (Oxford Nanopore Technologies), following manufacturer’s instructions.

### 2.4 Isolation and whole genome sequencing of *C. botulinum*

Confirmed positive enrichment cultures were streaked on Botulinum Selective Media (BSM) and incubated under anaerobic conditions to obtain isolated colonies. Representative colonies were screened by in-house multiplex PCR assays targeting BoNT/A, BoNT/B, *fld*B and *ntnh* genes. PCR-positive colonies were subsequently enriched overnight in TPGY broth. Genomic DNA was extracted from overnight cultures using silica-based spin columns, followed by library preparation with Nextera XT DNA Library Preparation Kit and sequencing using the MiSeq v2 Reagent Kit on the MiSeq platform (Illumina, CA, United States).

### 2.5 Sequencing analysis

The raw sequencing reads were checked using FastQC for Quality Control before *de novo* assembly using the SPAdes assembler. Species identity was confirmed using the Type (Strain) Genome Server (TYGS). Single nucleotide polymorphism (SNP) analysis was performed using the FDA CFSAN SNP Pipeline to assess genetic relatedness among isolates.

## 3 Results

Of the three lots of finished product initially tested as part of the outbreak response, 2 lots had presumptive positives (BoNT/A) – 4 of 12 samples from the first lot and 2 of 12 samples from the second lot (Table 1).

**Table 1:** Testing results for three lots of finished product.

Lot#	Sample ID	BoNT/A or BoNT/B real-time PCR	C. spp. (16S) real-time PCR	TAMS^a^ (CFU/g)	TAnMS^b^ (CFU/g)	SRC^c^ (CFU/g)
251261P2	1233392-001	BoNT/A only	Presumptive positive	20	ND^d^	ND
	1233392-002	Negative	Presumptive positive	ND	ND	ND
	1233392-003	Negative	Presumptive positive	ND	ND	ND
	1233392-004	BoNT/A only	Presumptive positive	40	ND	ND
	1233392-005	Negative	Presumptive positive	40	ND	ND
	1233392-006	Negative	Presumptive positive	20	ND	ND
	1233392-007	Negative	Presumptive positive	20	10	ND
	1233392-008	Negative	Presumptive positive	40	ND	ND
	1233392-009	BoNT/A only	Presumptive positive	20	ND	ND
	1233392-010	Negative	Presumptive positive	ND	10	ND
	1233392-011	Negative	Presumptive positive	ND	ND	ND
	1233392-012	BoNT/A only	Presumptive positive	20	10	ND
251131P2	1233393-001	Negative	Presumptive positive	20	20	ND
	1233393-002	Negative	Presumptive positive	40	10	ND
	1233393-003	Negative	Presumptive positive	10	10	ND
	1233393-004	Negative	Presumptive positive	ND	20	ND
	1233393-005	BoNT/A only	Presumptive positive	ND	ND	ND
	1233393-006	Negative	Presumptive positive	ND	10	ND
	1233393-007	Negative	Presumptive positive	20	ND	ND
	1233393-008	Negative	Presumptive positive	ND	ND	ND
	1233393-009	Negative	Presumptive positive	20	ND	ND
	1233393-010	Negative	Presumptive positive	50	ND	ND
	1233393-011	Negative	Presumptive positive	10	10	ND
	1233393-012	BoNT/A only	Presumptive positive	20	20	ND
243201P2	1233394-001	Negative	Presumptive positive	20	ND	ND
	1233394-002	Negative	Presumptive positive	20	ND	10
	1233394-003	Negative	Presumptive positive	10	ND	ND
	1233394-004	Negative	Presumptive positive	20	ND	ND
	1233394-005	Negative	Presumptive positive	20	10	ND
	1233394-006	Negative	Presumptive positive	40	30	ND
	1233394-007	Negative	Presumptive positive	10	10	ND
	1233394-008	Negative	Presumptive positive	50	10	ND
	1233394-009	Negative	Presumptive positive	10	10	ND
	1233394-010	Negative	Presumptive positive	20	ND	ND
	1233394-011	Negative	Presumptive positive	30	10	ND
	1233394-012	Negative	Presumptive positive	20	ND	ND

a TAMS, Thermal aerobic selection plating.

b TAnMS, Thermal anaerobic selection plating.

c Sulfite-reducing clostridia plating.

d Not detected.Real-time PCR positive for bont gene.

Of the 9 lots of base powder initially tested, 3 lots had presumptive positives – 2 of 8 samples from one lot and 1 of 8 samples each from the other two lots (Table 2).

**Table 2:** Testing results for nine lots of base powder.

Lot#	Tote	Mix	Sample ID	BoNT/A or BoNT/B real-time PCR	C. spp. (16S) real-time PCR	TAMS^a^ (CFU/g)	TAnMS^b^ (CFU/g)	SRC^c^ (CFU/g)
A	3	1	1233418-001	Negative	Presumptive positive	10	ND^d^	ND
	6	2	1233418-002	Negative	Presumptive positive	20	ND	ND
	10	3	1233418-003	Negative	Presumptive positive	10	ND	ND
	14	4	1233418-004	Negative	Presumptive positive	20	ND	ND
	18	5	1233418-005	Negative	Presumptive positive	10	ND	ND
	22	6	1233418-006	Negative	Presumptive positive	20	ND	ND
	26	7	1233418-007	Negative	Presumptive positive	20	ND	ND
B	3	1	1233418-008	Negative	Presumptive positive	30	10	ND
	6	2	1233418-009	Negative	Presumptive positive	10	ND	ND
	10	3	1233418-010	Negative	Presumptive positive	40	ND	ND
	14	4	1233418-011	Negative	Presumptive positive	20	ND	ND
	18	5	1233418-012	Negative	Presumptive positive	30	10	10
	22	6	1233418-013	Negative	Presumptive positive	20	10	ND
	26	7	1233418-014	Negative	Presumptive positive	40	ND	ND
C	3	1	1233418-015	Negative	Presumptive positive	10	ND	ND
	6	2	1233418-016	Negative	Presumptive positive	20	ND	ND
	10	3	1233418-017	Negative	Presumptive positive	20	10	ND
	14	4	1233418-018	Negative	Presumptive positive	ND	ND	ND
	18	5	1233418-019	Negative	Negative	10	10	ND
	22	6	1233418-020	Negative	Presumptive positive	ND	10	ND
	26	7	1233418-021	Negative	Presumptive positive	10	ND	ND
	30	8	1233418-022	Negative	Presumptive positive	10	ND	ND
D	3	1	1233418-023	Negative	Presumptive positive	10	30	ND
	6	2	1233418-024	Negative	Presumptive positive	10	ND	ND
	10	3	1233418-025	Negative	Presumptive positive	20	20	ND
	14	4	1233418-026	Negative	Presumptive positive	20	ND	ND
	18	5	1233418-027	Negative	Presumptive positive	30	20	ND
	22	6	1233418-028	Negative	Presumptive positive	10	ND	ND
	26	7	1233418-029	Negative	Presumptive positive	10	10	ND
	30	8	1233418-030	Negative	Presumptive positive	20	ND	ND
E	3	1	1233418-031	Negative	Presumptive positive	20	ND	ND
	6	2	1233418-032	BoNT/A only	Presumptive positive	20	10	ND
	10	3	1233418-033	Negative	Presumptive positive	30	10	10
	14	4	1233418-034	Negative	Presumptive positive	ND	ND	ND
	18	5	1233418-035	Negative	Presumptive positive	20	20	ND
	22	6	1233418-036	Negative	Presumptive positive	10	ND	ND
	26	7	1233418-037	Negative	Presumptive positive	10	ND	ND
	30	8	1233418-038	Negative	Presumptive positive	30	ND	ND
F	3	1	1233418-039	Negative	Presumptive positive	20	ND	ND
	6	2	1233418-040	Negative	Presumptive positive	10	20	ND
	10	3	1233418-041	Negative	Presumptive positive	10	10	ND
	14	4	1233418-042	Negative	Presumptive positive	20	10	ND
	18	5	1233418-043	Negative	Presumptive positive	ND	ND	ND
	22	6	1233418-044	Negative	Presumptive positive	20	10	ND
	26	7	1233418-045	Negative	Presumptive positive	20	ND	ND
	30	8	1233418-046	Negative	Presumptive positive	30	ND	ND
G	3	1	1233418-047	Negative	Presumptive positive	10	ND	ND
	5	2	1233418-048	Negative	Presumptive positive	ND	ND	ND
	7	3	1233418-049	Negative	Presumptive positive	ND	ND	ND
	10	4	1233418-050	Negative	Presumptive positive	10	10	ND
	12	5	1233418-051	Negative	Presumptive positive	ND	ND	ND
	16	6	1233418-052	Negative	Presumptive positive	10	10	ND
	20	7	1233418-053	Negative	Presumptive positive	10	ND	ND
	24	8	1233418-054	Negative	Presumptive positive	20	ND	ND
H	3	1	1233418-055	Negative	Presumptive positive	10	10	ND
	6	2	1233418-056	Negative	Presumptive positive	10	ND	ND
	10	3	1233418-057	Negative	Presumptive positive	10	ND	ND
	14	4	1233418-058	Negative	Presumptive positive	ND	10	ND
	18	5	1233418-059	Negative	Presumptive positive	40	ND	ND
	22	6	1233418-060	BoNT/A only	Presumptive positive	20	ND	ND
	26	7	1233418-061	BoNT/A only	Presumptive positive	30	10	ND
	30	8	1233418-062	Negative	Presumptive positive	20	ND	ND
I	3	1	1233418-063	Negative	Presumptive positive	10	ND	ND
	6	2	1233418-064	Negative	Presumptive positive	30	ND	ND
	10	3	1233418-065	Negative	Presumptive positive	20	ND	ND
	14	4	1233418-066	BoNT/A only	Presumptive positive	ND	20	ND
	18	5	1233418-067	Negative	Presumptive positive	ND	ND	ND
	22	6	1233418-068	Negative	Presumptive positive	20	ND	ND
	26	7	1233418-069	Negative	Presumptive positive	30	ND	ND
	30	8	1233418-070	Negative	Presumptive positive	20	10	ND

a TAMS, Thermal aerobic selection plating.

b TAnMS, Thermal anaerobic selection plating.

c Sulfite-reducing clostridia plating.

d Not detected.Real-time PCR positive for bont gene.

All six presumptive positives from finished product (Table 1) and two presumptive positives from base powder confirmed positive for *C. botulinum* by amplicon sequencing of the neurotoxin genes.

Confirmed positive enrichment samples were streaked for isolation. Four colonies from lot #1233393-005 and one colony from lot #1233392-004 of finished product were isolated. One colony from lot #1233418-066 of base powder was also isolated.

Bacteria from isolated colonies were subjected to whole genome sequencing and compared to each other as well as four whole genome sequences from NCBI using SNP analysis (Table 3). The four isolates taken from finished product lot no. 1233393-005 are identical to each other but very different (>400 SNPs) as compared to the isolate from finished product lot no. 1233392-004. The two isolates from base powder are identical in sequence to the four isolates from finished product lot #1233393-005. None of the isolates were a genetic match to the NCBI strains listed in the table as well as in a more comprehensive search of all available genome sequences in NCBI.

**Table 3:** SNP analysis of *C. botulinum* isolates from finished product and base powder, compared against four whole genome sequences in NCBI.

SNP analysis	NCBI 1	NCBI 2	NCBI 3	NCBI 4	1233393-005 A	1233393-005 B	1233393-005 C	1233393-005 D	1233392-004	1233418-066 A	1233418-066 B
NCBI 1	0	313	88	176	417	418	415	417	83	407	415
NCBI 2	313	0	325	372	321	322	321	321	322	314	319
NCBI 3	88	325	0	190	433	434	431	433	55	422	431
NCBI 4	176	372	190	0	475	476	474	475	185	465	473
1233393-005 A	417	321	433	475	0	1	0	0	429	4	4
1233393-005 B	418	322	434	476	1	0	1	1	430	5	5
1233393-005C	415	321	431	474	0	1	0	0	427	4	4
1233393-005 D	417	321	433	475	0	1	0	0	429	4	4
1233392-004	83	322	55	185	429	430	427	429	0	418	427
1233418-066 A	407	314	422	465	4	5	4	4	418	0	0
1233418-066 B	415	319	431	473	4	5	4	4	427	0	0

NCBI 1, SRR2082799; NCBI 2, SRR24495897; NCBI 3, SRR35098139; NCBI 4, SRR35098172. 1233393-005A-D from finished product (NCBI SRR36912166, CFSAN145336, SRR36912165, SRR36912164); 1233392-004 from finished product (NCBI SRR3692272); 1233418-066 from base powder (NCBI SRR36912271). Less than 10 SNPs (genetically identical).

## 4 Discussion

The 2025 United States infant botulism outbreak, linked to a brand of infant formula with 51 cases reported at the time of this submission, is a watershed event. It shifts the risk assessment for *C. botulinum* in PIF from a theoretical hazard to one that is “reasonably likely to occur.” Currently, PIF and specific ingredients, such as dairy materials, are not subjected to pasteurization processes capable of delivering a 6-log reduction of heat-resistant *C. botulinum* spores.

Furthermore, testing for *C. botulinum* is extremely challenging, particularly with regards to culture confirmation. Real-time PCR screening of sample enrichments revealed that non-target clostridial species can be present at concentrations six orders of magnitude (10^6^) higher than *C. botulinum*. This resulted in Cq values more than 20 cycles earlier than the target (data not shown). In this study, examining more than 1,500 colonies was required to find a single positive *C. botulinum* colony, highlighting the critical importance of molecular approaches for confirmation.

Following the outbreak and subsequent recall, several groups promoted SRC testing as a primary control measure. However, our evidence highlights significant risks in this approach. The recovery of *C. botulinum* from finished products – confirmed by real-time PCR and genomic analysis – demonstrates the limitations of indicator-based screening. Notably, these findings occurred in samples where SRC were not detected, indicating that SRC enumeration did not reliably correlate with the presence of toxigenic *C. botulinum*. Similar shortcomings of indicator organisms have been noted previously (Hauschild and Dodds, 1993; International Commission on Microbiological Specifications for Foods (ICMSF), 2014).

This observation is consistent with the biological heterogeneity of the species; while sulfite reduction is common among clostridia, it is not a universal phenotype for all toxigenic *C. botulinum* strains. Phenotypic expression is heavily influenced by strain-specific traits and assay conditions (Lindström and Korkeala, 2006; Peck, 2009). Consequently, while SRC-based methods are useful for assessing general hygiene, they have inherent limitations as predictors of *C. botulinum* (International Commission on Microbiological Specifications for Foods (ICMSF), 2014). These limitations may stem from high effective limits of detection (≤10 CFU/g) and inefficient spore germination or outgrowth on selective agar (Hauschild and Dodds, 1993).

While these constraints may be inconsequential in high-level contamination, they are critical at low levels observed here. Even low numbers of *C. botulinum* pose a significant risk to infants, who lack the established gut microbiota necessary to suppress colonization (Centers for Disease Control and Prevention (CDC), 1998; Lindström and Korkeala, 2006; Harris and Dabritz, 2024). The tiered analytical approach employed in this study – combining anaerobic enrichment with molecular screening – enabled the identification of samples requiring further investigation and guided targeted isolation efforts. Real-time PCR provided a sensitive screening tool, while amplicon sequencing and WGS provided the specificity and resolution necessary to link the outbreak definitively to the product (ISO/TS 17919:2013, 2013; Lindström and Korkeala, 2006; U.S. Food and Drug Administration (FDA), 2025).

### 4.1 Implications for industry

At the time of writing, the root cause investigation remains ongoing to determine likely sources of *C. botulinum* entry into the supply chain. The 2025 infant botulism outbreak – with 51 cases reported by the CDC as of publication – represents the first outbreak linked to PIF in which *C. botulinum* was detected in unopened finished product containers. This outbreak fundamentally changes the standing assumptions in PIF manufacturing: that *C. botulinum* is not an organism of concern requiring specific preventative controls.

The most significant consequence of this outbreak for the PIF industry is that *C. botulinum* can no longer be classified an unforeseen hazard. This necessitates an immediate reassessment of hazard analysis across the industry. Furthermore, these results highlight a critical consideration for industry testing programs: enumeration of SRC should be interpreted strictly as an indicator of general anaerobic spore presence rather than a reliable surrogate for *C. botulinum* detection. Reliance on SRC results alone can lead to false-negative conclusions; the test lacks the necessary sensitivity or specificity to serve as a proxy for toxigenic *C. botulinum*.

Incorporation of targeted molecular screening for botulinum neurotoxin genes – particularly within a tiered testing framework for high-risk products like PIF – can significantly enhance detection sensitivity while maintaining alignment with modern preventive control principles. Such an approach supports earlier detection, more informed risk-management decisions, and improved confidence in food safety assessments involving *C. botulinum*.

## Data Availability

The datasets presented in this study can be found in online repositories. The names of the repository/repositories and accession number(s) can be found at: https://www.ncbi.nlm.nih.gov/, NCBI 1 = SRR2082799; NCBI 2 = SRR24495897; NCBI 3 = SRR35098139; NCBI 4 = SRR35098172 1233393-005A-D (NCBI SRR36912166, CFSAN145336, SRR36912165, SRR36912164); 1233392-004 (NCBI SRR3692272); 1233418-066 (NCBI SRR36912271).

## Footnotes

1
https://www.fda.gov/food/outbreaks-foodborne-illness/outbreak-investigation-infant-botulism-infant-formula-november-2025
